# New insight into telomere maintenance

**DOI:** 10.18632/aging.100147

**Published:** 2010-05-14

**Authors:** Ryan T.Y. Wu, Wen-Hsing Cheng

**Affiliations:** Department of nutrition and Food Science, University of Maryland, College Park, MD, 20742, USA

**Keywords:** Kusumoto-Matsuo et al. Cooperation of DNA-PKcs and WRN helicase in the maintenance of telomeric D-loops. Aging. 2010; 2:255-256

Last year is the year of telomere. The Nobel Prize in physiology or medicine 2009 was awarded to Elizabeth Blackburn, Carol Greider, and Jack Szostak for their seminal discovery of telomere and telomerase. Telomere is a compact DNA-protein structure at the end of chromosome, comprised of TTAGGG repeats in humans and proteins associated with the telomeric sequence. When DNA replication starts, both strands of DNA are synthesized in opposite directions. The leading strand moves continuously, while the lagging strand is generated discontinuously. There is a need of a RNA primer, known as Okazaki fragment, to initiate telomere synthesis on lagging strand. The primer has an intrinsic nature of being degraded, resulting in a loss of some repetitive telomere sequence after the completion of each round of DNA replication. Telomere is one of the key determinants of lifespan, and telomere erosion to a critically short stage limits replicative lifespan of cultured cells, a phenomenon known as Hayflick limit or replicative senescence [1].

Nonetheless, telomere length is not the only contributor to replicative senescence. The length and structure of telomere are tightly controlled by many telomere-associated proteins, mutations in some of which result in premature aging syndromes. In particular, the *WRN *gene is mutated in Werner syndrome, characterized by accelerated aging phenotypes after puberty. The WRN protein exhibits helicase and exonuclease activities that are critical for DNA metabolism by unwinding or processing DNA substrates. WRN preferentially binds DNA  structures  reminiscent of  intermediates during DNA replication [2], and the WRN helicase activity maintains telomere homeostasis only during DNA replication [3]. A previous study showed that WRN can form a complex with DNA-PK, the key enzyme in the non-homologous end-joining pathway of DNA double strand break repair [4]. Loss-of-function in the catalytic activity of DNA-PK (DNA-PK_cs_) results in chromosome end-to-end fusion [5]. Strikingly, mice deficient in either DNA-PK_cs _or WRN alone do not show aging phenotypes unless they are under a short telomere background. Consistent with this notion, Kusumoto-Matsuo et al. have now identified a key link between the WRN helicase and DNA-PK_cs_ at telomeres for the maintenance of telomeric D-loop structure [6]. They provided evidence that DNA-PKcs can specifically stimulate WRN helicase and prevent telomeric D-loop from exonuclease digestion *in vitro* and *in vivo.* Moreover, they showed that the telomere repetitive sequence, known as G-tail, is shorter in DNA-PKcs or WRN deficient cells than in normal cells, and these defects can be reversed by overexpressing WRN in DNA-PKcs deficient cells. Altogether, DNA-PKcs can help WRN to unwind telomere D-loop and protect the DNA from exonuclease actions.

The desire of deciphering longevity is likely to drive scientists to continuously explore telomere maintenance. Understanding the cross-talks between the proteins associated with telomere will one day provide the mechanistic basis for telomere-associated aging phenotypes. Of note, Mre11 was found to exhibit a protective role in generating 3' overhang at telomeres to avoid triggering DNA repair mechanisms [7]. The interaction between DNA-PKcs and WRN might act as an upstream event of telomeric D-loop processing and the front line of telomere protection.
